# Supplementary material for: Early spatiotemporal dynamics of navigational affordance coding in the dorsal visual cortex
Source: Nat Commun. 2026 Jan 8;17:1361. doi: 10.1038/s41467-025-68111-7 (PMC12876848; doi:10.1038/s41467-025-68111-7)
Supplement: Supplementary file 1 — Reporting Summary [file 41467_2025_68111_MOESM1_ESM.pdf]

nature portfolio

Corresponding author(s): Edward Silson (ed.silson@ed.ac.uk)

Last updated by author(s): Dec 9, 2025

Reporting Summary

Nature Portfolio wishes to improve the reproducibility of the work that we publish. This form provides structure for consistency and transparency in reporting. For further information on Nature Portfolio policies, see our Editorial Policies and the Editorial Policy Checklist.

Statistics

For all statistical analyses, confirm that the following items are present in the figure legend, table legend, main text, or Methods section.

n/a	Confirmed	
☐	☒	The exact sample size (*n*) for each experimental group/condition, given as a discrete number and unit of measurement
☐	☒	A statement on whether measurements were taken from distinct samples or whether the same sample was measured repeatedly
☐	☒	The statistical test(s) used AND whether they are one- or two-sided<br>*Only common tests should be described solely by name; describe more complex techniques in the Methods section.*
☐	☒	A description of all covariates tested
☐	☒	A description of any assumptions or corrections, such as tests of normality and adjustment for multiple comparisons
☐	☒	A full description of the statistical parameters including central tendency (e.g. means) or other basic estimates (e.g. regression coefficient) AND variation (e.g. standard deviation) or associated estimates of uncertainty (e.g. confidence intervals)
☐	☒	For null hypothesis testing, the test statistic (e.g. *F*, *t*, *r*) with confidence intervals, effect sizes, degrees of freedom and *P* value noted<br>*Give P values as exact values whenever suitable.*
☐	☒	For Bayesian analysis, information on the choice of priors and Markov chain Monte Carlo settings
☒	☐	For hierarchical and complex designs, identification of the appropriate level for tests and full reporting of outcomes
☐	☒	Estimates of effect sizes (e.g. Cohen's *d*, Pearson's *r*), indicating how they were calculated

Our web collection on statistics for biologists contains articles on many of the points above.

Software and code

Policy information about availability of computer code

Data collection	Across all experiments (fMRI, MEG & Behaviour) visual stimuli were presented using PsychoPy (2024.2.3). fMRI data were collected using Siemens 3T Prisma scanner and 32-channel head coil. MEG data was recorded on a 248-channel 4D Neuroimaging Magnes 3600 MEG with electronics upgraded by York Instruments Ltd.
Data analysis	The analysis pipeline consisted of different steps:<br>- Preprocessing of MRI/fMRI data were performed in AFNI (version 24_3_10)<br>- GLM analyses were performed in AFNI (version 24_3_10).<br>- Anatomical segmentation was performed using FreeSurfer.<br>- MEG analyses were performed using Matlab (version 2025_b)<br>- Searchlight analyses were performed using the CoSMoMVPA toolbox implemented in Matlab.<br>- Frequentist analyses were performed with Matlab<br>- Bayesian analyses were performed with Matlab using the follwoing toolbox https://github.com/LinaTeichmann1/BFF_repo<br>- Behavioural data were analysed using R Studio (version 2024.12.0)

For manuscripts utilizing custom algorithms or software that are central to the research but not yet described in published literature, software must be made available to editors and reviewers. We strongly encourage code deposition in a community repository (e.g. GitHub). See the Nature Portfolio guidelines for submitting code & software for further information.

Data

Policy information about availability of data

All manuscripts must include a data availability statement. This statement should provide the following information, where applicable:

- Accession codes, unique identifiers, or web links for publicly available datasets
- A description of any restrictions on data availability
- For clinical datasets or third party data, please ensure that the statement adheres to our policy

Anonymised pre-processed fMRI, MEG and behavioural data are available via the Open Science Framework along with analysis code ((https://doi.org/10.17605/OSF.IO/PQ2M3)). Raw fMRI and MEG data will be made available upon request.

Research involving human participants, their data, or biological material

Policy information about studies with human participants or human data. See also policy information about sex, gender (identity/presentation), and sexual orientation and race, ethnicity and racism.

Reporting on sex and gender	The findings do not apply to one sex or gender, and neither were considered in study design or analyses. Sex was determined based on self-reporting.
Reporting on race, ethnicity, or other socially relevant groupings	Racial or ethnic characteristics were not recorded.
Population characteristics	Our population had no particular characteristic in terms of demographics.
Recruitment	An opportunity sampling strategy was used for both the fMRI/MEG and behavioural experiments. Participants were recruited from the University populations of both the University of York and the University of Edinburgh.
Ethics oversight	Ethical approval was provided by both the York Neuroimaging centre (University of York) and the School of Philosophy, Psychology and Language Sciences (University of Edinburgh). All participants gave informed consent.

Note that full information on the approval of the study protocol must also be provided in the manuscript.

Field-specific reporting

Please select the one below that is the best fit for your research. If you are not sure, read the appropriate sections before making your selection.

☐ Life sciences ☒ Behavioural & social sciences ☐ Ecological, evolutionary & environmental sciences

For a reference copy of the document with all sections, see nature.com/documents/nr-reporting-summary-flat.pdf

Behavioural & social sciences study design

All studies must disclose on these points even when the disclosure is negative.

Study description	This is a quantitative study using fMRI, MEG and behavioural testing.
Research sample	The sample consists of two cohorots fo healthy volunteers. Cohort 1, recruited from the University of York consists of 14 particiapnts (10 female) with a mean age of 24.43 (+/- 4.85) years. Cohort 2, recruited from the University of Edinburgh consists of 72 (52 female) participants with a mean age of 28.51 (+/- 4.10) years.
Sampling strategy	An opportunity sampling strategy was used for both the fMRI/MEG and behavioural experiments. Participants were recruited from the University populations of both the University of York and the University of Edinburgh.
Data collection	The delivery system used for the visual stimulus in fMRI scans was a ViewPixx projector which projected the stimulus onto a custom-made acrylic screen. In MEG the stimuli were presented using a ViewPixx Projector which projected the stimulus onto a screen in the MEG suite. Visual stimuli for the behavioural experiment were presented via a HP EliteDesk monitor (resolution: 1920 × 1080 pixels; refresh rate: 60 Hz; visible display size: 531.36 mm × 298.89 mm). fMRI data were collected using Siemens 3T Prisma scanner and 32-channel head coil. MEG data was recorded on a 248-channel 4D Neuroimaging Magnes 3600 MEG with electronics upgraded by York Instruments Ltd.
Timing	04/2023 to 07/2024
Data exclusions	No data were excluded from the analyses.
Non-participation	No participants dropped out of the experiments.
Randomization	Participants were not allocated into experimental groups.

Reporting for specific materials, systems and methods

We require information from authors about some types of materials, experimental systems and methods used in many studies. Here, indicate whether each material, system or method listed is relevant to your study. If you are not sure if a list item applies to your research, read the appropriate section before selecting a response.

Materials & experimental systems

n/a	Involved in the study
☒	☐ Antibodies
☒	☐ Eukaryotic cell lines
☒	☐ Palaeontology and archaeology
☒	☐ Animals and other organisms
☒	☐ Clinical data
☒	☐ Dual use research of concern
☒	☐ Plants

Methods

n/a	Involved in the study
☒	☐ ChIP-seq
☒	☐ Flow cytometry
☐	☒ MRI-based neuroimaging

Plants

Seed stocks

Report on the source of all seed stocks or other plant material used. If applicable, state the seed stock centre and catalogue number. If plant specimens were collected from the field, describe the collection location, date and sampling procedures.

Novel plant genotypes

Describe the methods by which all novel plant genotypes were produced. This includes those generated by transgenic approaches, gene editing, chemical/radiation-based mutagenesis and hybridization. For transgenic lines, describe the transformation method, the number of independent lines analyzed and the generation upon which experiments were performed. For gene-edited lines, describe the editor used, the endogenous sequence targeted for editing, the targeting guide RNA sequence (if applicable) and how the editor was applied.

Authentication

Describe any authentication procedures for each seed stock used or novel genotype generated. Describe any experiments used to assess the effect of a mutation and, where applicable, how potential secondary effects (e.g. second site T-DNA insertions, mosiacism, off-target gene editing) were examined.

Magnetic resonance imaging

Experimental design

Design type: Event-related, on/off block and population recceptive field modelling.

Design specifications: Event-related and block design

Behavioral performance measures: Orthogonal task accuracy

Acquisition

Imaging type(s): Multi-echo fMRI

Field strength: 3.0T

Sequence & imaging parameters: All functional scans consisted of 48 multiband-multiecho EPI slices (acceleration factor = 2, TEs = 14.6, 32.83, 51.06ms) within a FOV of 240x240mm with 2.7 mm isotropic voxels (TR = 2000 ms, flip angle = 150°).

Area of acquisition: Brain

Diffusion MRI ☐ Used ☒ Not used

Preprocessing

Preprocessing software: AFNI

Normalization: Data were analysed in the participant native space

Normalization template: Data were analysed in the participant native space

Noise and artifact removal: De-spiking, de-trending and motion correction

Volume censoring: Brain automask (3dAutomask AFNI command)

Statistical modeling & inference

Model type and settings	General Linear Model, Searchlight
Effect(s) tested	For the OPA localiser, a GLM contrast of Scenes > Faces was computed. This produced a t-statistic for each voxel/node. For the navigational affordance data, a GLM contrast was computed for each stimulus versus baseline. This produced a t-statistic for each voxel/node. For the searchlight analyses, a partial pearson's correlation coefficient was computed for each voxel/node.

Specify type of analysis: ☐ Whole brain ☐ ROI-based ☒ Both

Anatomical location(s)	OPA ROIs were defined in each participant using the contrast of Scenes > Faces ($p<0.001$). The Borders of retinotopic maps were defined using a probabilistic atlas.
Statistic type for inference (See Eklund et al. 2016)	F-stat, t-stat, Pearson's r
Correction	FDR

Models & analysis

n/a	Involved in the study
☒	☐ Functional and/or effective connectivity
☒	☐ Graph analysis
☐	☒ Multivariate modeling or predictive analysis

Multivariate modeling and predictive analysis	Representational similarity analysis
